# MRI BrainAGE demonstrates increased brain aging in systemic lupus erythematosus patients

**DOI:** 10.3389/fnagi.2023.1274061

**Published:** 2023-10-20

**Authors:** Grégory Kuchcinski, Theodor Rumetshofer, Kristoffer A. Zervides, Renaud Lopes, Morgan Gautherot, Jean-Pierre Pruvo, Anders A. Bengtsson, Oskar Hansson, Andreas Jönsen, Pia C. Maly Sundgren

**Affiliations:** ^1^Division of Diagnostic Radiology, Department of Clinical Sciences, Skåne University Hospital, Lund University, Lund, Sweden; ^2^Lund University BioImaging Centre, Lund University, Lund, Sweden; ^3^Inserm, CHU Lille, U1172 – LilNCog – Lille Neuroscience & Cognition, Univ. Lille, Lille, France; ^4^Division of Logopedics, Phoniatrics and Audiology, Department of Clinical Sciences, Lund University, Lund, Sweden; ^5^Division of Rheumatology, Department of Clinical Sciences, Skåne University Hospital, Lund University, Lund, Sweden; ^6^Univ. Lille, CNRS, Inserm, CHU Lille, Institut Pasteur de Lille, Lille, France; ^7^Clinical Memory Research Unit, Lund University, Lund, Sweden; ^8^Memory Clinic, Skåne University Hospital, Malmö, Sweden

**Keywords:** systemic lupus erythematosus, brain, aging, deep learning, magnetic resonance imaging

## Abstract

**Introduction:**

Systemic lupus erythematosus (SLE) is an autoimmune connective tissue disease affecting multiple organs in the human body, including the central nervous system. Recently, an artificial intelligence method called BrainAGE (Brain Age Gap Estimation), defined as predicted age minus chronological age, has been developed to measure the deviation of brain aging from a healthy population using MRI. Our aim was to evaluate brain aging in SLE patients using a deep-learning BrainAGE model.

**Methods:**

Seventy female patients with a clinical diagnosis of SLE and 24 healthy age-matched control females, were included in this post-hoc analysis of prospectively acquired data. All subjects had previously undergone a 3 T MRI acquisition, a neuropsychological evaluation and a measurement of neurofilament light protein in plasma (NfL). A BrainAGE model with a 3D convolutional neural network architecture, pre-trained on the 3D-T1 images of 1,295 healthy female subjects to predict their chronological age, was applied on the images of SLE patients and controls in order to compute the BrainAGE. SLE patients were divided into 2 groups according to the BrainAGE distribution (high vs. low BrainAGE).

**Results:**

BrainAGE *z*-score was significantly higher in SLE patients than in controls (+0.6 [±1.1] vs. 0 [±1.0], *p* = 0.02). In SLE patients, high BrainAGE was associated with longer reaction times (*p* = 0.02), lower psychomotor speed (*p* = 0.001) and cognitive flexibility (*p* = 0.04), as well as with higher NfL after adjusting for age (*p* = 0.001).

**Conclusion:**

Using a deep-learning BrainAGE model, we provide evidence of increased brain aging in SLE patients, which reflected neuronal damage and cognitive impairment.

## 1. Introduction

Systemic lupus erythematosus (SLE) is an autoimmune connective tissue disease that affects 0.1% of the general population, with a large female predominance. Neuropsychiatric symptoms are commonly observed in individuals with SLE, with reported frequencies ranging from 20 to 95%, depending on the classification criteria used (Brey et al., 2002). These symptoms have been associated with increased morbidity and mortality and decreased quality of life (Brey et al., 2002). The array of neuropsychiatric symptoms is diverse, encompassing conditions such as headaches, epilepsy, focal neurological deficits, mood disorders, and psychosis (Brey et al., 2002). Among these, cognitive dysfunction is particularly prevalent, affecting approximately 75% of patients (Leslie and Crowe, 2018). The pathophysiology of these symptoms remains widely debated.

Brain abnormalities observed in SLE patients are frequent and heterogeneous. Beyond the vascular and/or inflammatory lesions identified through MRI (Jennings et al., 2004) and histopathological studies (Hanly et al., 1992; Cohen et al., 2017), mounting evidence suggests an ongoing neurodegenerative process. Histological examinations have highlighted substantial and widespread neuronal loss among patients displaying neuropsychiatric symptoms (Ercan et al., 2016). Initial longitudinal imaging studies have confirmed progressive reductions in brain volume, affecting both white and gray matter, over relatively short time spans (19 months) (Appenzeller et al., 2007). The neurodegenerative nature of these changes has been further supported by elevated levels of CSF and plasma biomarkers associated with ongoing neuronal damage (neurofilament light protein [NfL] and glial fibrillary acidic protein) (Trysberg et al., 2003, 2004; Lauvsnes et al., 2022), even among patients without neuropsychiatric symptoms. Interestingly, this phenomenom has also been observed at the cellular level, with the accumulation of senescent neural cells in the hippocampus of murine models of SLE (MRL/Ipr mice) (Saito et al., 2021).

Nonetheless, these lesions lack specificity, exhibiting a continuum across patients with and without neuropsychiatric symptoms. In approximately 60% of cases, symptoms may be independent of brain damage, and instead attributed to chronic pain, altered sleep quality or corticosteroid therapy (Magro-Checa et al., 2016). The broad range of symptoms presented and the absence of a reliable biomarker therefore make diagnosis and management challenging in a large number of cases (Kalinowska-Lyszczarz et al., 2018).

Recently, an artificial intelligence method, called BrainAGE (Brain Age Gap Estimation), has been developed to measure the deviation in brain aging within a cohort of patients experiencing cognitive decline, in comparison to a healthy population without cognitive or psychiatric disorders (Franke and Gaser, 2019). The approach involves training a model to predict the age of healthy individuals based on MR images of their brain. Subsequently, this model can be applied to a group of patients with cognitive and/or psychiatric disorders, aiming to reveal the discrepancy between the predicted age generated by the algorithm and the actual age of the patient - termed BrainAGE score. A BrainAGE score near 0 indicates typical brain aging, while a BrainAGE score significantly higher than 0 suggests increased brain aging. This quantitative, cross-diagnostic marker effectively reflects the extent of disease-related structural changes. Notably, BrainAGE is sensitive to volume loss related to cerebral atrophy, but also to signal changes induced, for instance, by white matter lesions (Bretzner et al., 2023).

Our main objective was to evaluate brain aging in SLE by using a deep-learning BrainAGE model within an established cohort of extensively characterized SLE patients and matched healthy controls (Cannerfelt et al., 2018; Langensee et al., 2022; Zervides et al., 2022b). Our secondary objectives were to identify neuropsychological correlates and clinical factors contributing to increased brain aging.

## 2. Materials and methods

### 2.1. Population

The present study is a cross-sectional post-hoc MRI analysis of a well described single-center prospective SLE cohort (Cannerfelt et al., 2018; Langensee et al., 2022; Zervides et al., 2022b). The study cohort was approved by the regional ethics committee (reference #2012/254, #2012/677, #2014/778). The study was conducted in accordance with the Declaration of Helsinki. Written informed consent was obtained from the patients prior to data collection.

Between 2013 and 2016, consecutive prevalent patients with a diagnosis of SLE, attending the Department of Rheumatology, were prospectively enrolled. Inclusion criteria were as follows: diagnostic of SLE meeting at least 4 criteria of the American College of Rheumatology (ACR) classification (Tan et al., 1982), female gender, age between 18 and 55 years and right-handedness. Patients with any contra-indication to MRI were excluded (e.g., pace-maker, pregnancy).

During the same time period, female control subjects within the same age range, free of autoimmune, neurological or psychiatric disorders were recruited in a control group among health care workers and university employees at our institution. Seventy-one patients and 25 age-matched controls constituted the cohort population. Two participants (1 SLE patient and 1 healthy control) were secondarily excluded from the present analysis because of they had a focal brain lesion (meningioma, *n* = 1; cystic lesion, *n* = 1).

### 2.2. Clinical and neuropsychological evaluation

All patients were evaluated by a rheumatologist and a neurologist, as previously described (Zervides et al., 2022b). Neuropsychiatric manifestations were defined according to the Systemic Lupus International Collaborating Clinics (SLICC) attribution models (more stringent “SLICC A” and less stringent “SLICC B”) (Hanly et al., 2007). Organ damage was recorded according to the SLICC/ACR damage index (SLICC/ACR-DI) (Gladman et al., 1996), and disease activity was assessed using the Systemic Lupus Erythematosus Disease Activity Index 2000 (SLEDAI-2 K) (Gladman et al., 2002).

All subjects underwent neuropsychological testing by a neuropsychologist using a standardized neurocognitive test battery (CNS Vital Signs; Gualtieri and Johnson, 2006), described elsewhere (Langensee et al., 2022). CNS Vital Signs has been previously tested and validated in SLE patients (Langensee et al., 2022), as well in traumatic brain injury, dementia and attention deficit hyperactivity disorder (Gualtieri and Johnson, 2006; Littleton et al., 2015). Briefly, seven established tests (verbal memory, visual memory, finger tapping, symbol digit coding, Stroop, shifting attention and continuous performance) were used to compute age-adjusted scores in several cognitive domains. For the purpose of this study, we recorded composite memory, psychomotor speed, reaction time, complex attention and cognitive flexibility. These scores have a mean of 100 and a standard deviation of 15 in a normative sample provided by the CNS Vital Signs software. A psychologist, who remained present for the duration of the session, tested participants individually. A brief oral introduction by the psychologist, explaining the testing procedure was given to each participant. Following this, they completed the test battery independently according to the instructions that were given on the screen prior to each of the tests (Langensee et al., 2022). Additional clarifications were provided when requested. Each of the individual tests had to be performed for a predetermined amount of time and most tests were in turn timed internally, so that a response had to be given within the provided time window. Completing the entire test battery took approximately 30 min (variations occurred due to participants taking varying amounts of time to read the instructions).

Psychomotor speed was evaluated using two tests, the Finger Taping Test and the Symbol Digit Coding test (Gualtieri and Johnson, 2006). For the Finger Taping test, subjects are asked to press a space bar with left and right index finger as many times as they can in 10 s. For the Symbol Digit Coding test, the subject is given a training session to learn how to link numbers to digits. During the test session, the subject types in the number that corresponds to each symbol that is presented on the screen during 120 s. Psychomotor speed is a composite score calculated automatically by the software by adding the total of right and left taps from the Finger Taping Test and the total of correct responses during the Symbol Digit Test.

Reaction time was evaluated during a Stroop Test (Gualtieri and Johnson, 2006). During the first part of the test, the words RED, YELLOW, BLUE, and GREEN appear on the screen, printed in color. The subject is asked to press the space bar when the color of the word matches what the word says. This generates a complex reaction time score. During the second part of the test, the subject is asked to press the space bar when the color of the word does not match what the word says. This part also generates a complex reaction time score, called the “color-word reaction time.” Averaging the two complex reaction time scores from the Stroop test generates a composite score for “reaction time.”

### 2.3. Laboratory analyses

Serum and plasma samples were obtained from all patients within two weeks before or after MRI. Routine biochemical and immunological analyses were performed at the Departments of Laboratory Medicine and Immunology, including measurements of serum levels of complement factors, anti-double-stranded DNA antibodies, and antiphospholipid antibodies (including serum IgG anti-cardiolipin antibodies, serum IgG anti-beta-2-glycoprotein-1 antibodies and Lupus Anticoagulant). The concentrations of protein S100A8/A9 in serum were measured with the Bühlmann MRP8/14 ELISA kit, Switzerland, according to the manufacturers’ instructions (Zervides et al., 2022b). Plasma NfL concentrations were measured using a single-molecule array (Quanterix; Billerica, MA) and the commercially available NfL assay was utilized (NF-light™ # 103186) (Zervides et al., 2022a).

### 2.4. Neuroimaging

#### 2.4.1. MRI acquisitions

MRI acquisitions were performed on a 3T MRI (Siemens MAGNETOM Skyra, Erlangen, Germany), using a 20-channel head coil. The protocol included a 3D-T1 MPRAGE (magnetization-prepared rapid gradient-echo) (voxel size = 1 mm^3^ isotropic, matrix = 256 × 256 × 176, TE = 2.54 ms, TR = 1900 ms, TI = 900 ms, flip angle = 9°) and a FLAIR sequence (voxel size = 0.7 × 0.7 × 3 mm, matrix = 280 × 320 × 33, TE = 81 ms, TR = 9000 ms, TI = 2500 ms, flip angle = 150°).

#### 2.4.2. MRI visual analysis and white-matter hyperintensities segmentation

All scans were visually evaluated by a board certified neuroradiologist. A complete description of morphological abnormalities in this cohort has been reported elsewhere (Cannerfelt et al., 2018). For the purpose of this study, we reported only vascular lesions susceptible to influence BrainAGE prediction: previous ischemic or hemorrhagic lesions and white matter hyperintensities. White matter hyperintensities were automatically segmented by the lesion growth algorithm implemented in the Lesion Segmentation Toolbox (LST, version 2.0.14),^1^ using 3D-T1 and FLAIR images (Schmidt et al., 2012).

#### 2.4.3. BrainAGE preprocessing

Minimal preprocessing steps were applied on 3D-T1 brain images. First, images were corrected for magnetic field inhomogeneity effects and skull-stripped using VolBrain software version 1.0 (RRID:SCR_021020)^3^ (Manjón and Coupé, 2016). Brain extractions were systematically checked, and manual corrections were performed by a neuroradiologist, when deemed necessary. Then, preprocessed 3D-T1 images were linearly registered and resampled into the MNI space using SPM software version 12 (RRID:SCR_007037). Finally, intensity normalization was performed using min–max normalization. Gray matter, white matter and CSF volumes were automatically calculated using VolBrain software.

#### 2.4.4. Deep-learning BrainAGE model

For the prediction of chronological age based on brain images, our model was based on a 3D convolutional neural network architecture, as previously published (Gautherot et al., 2021). For the purpose of this study, the model was trained and validated on a dataset of brain MRI volumes from 1503 healthy female participants between 18 and 70 years (training *n* = 1,295, validation *n* = 208).

The training dataset was constituted of data compiled from several publicly available sources: IXI (Information eXtraction from Images),^4^ HCP (Human Connectome Project),^5^ OBRE (Center of Biomedical Research Excellence),^6^ MCIC (Mind Clinical Imaging Consortium),^7^ NMorphCH (Neuromorphometry by Computer Algorithm Chicago),^8^ NKI-RS (Enhanced Nathan Kline Institute-Rockland Sample),^9^ PPMI (Parkinson’s Progression Markers Initiative)^10^ and ADNI (Alzheimer’s Disease Neuroimaging Initiative).^11^

The model input was preprocessed 3D-T1 images with dimensions of 182 × 218 × 182 voxels. The model was based on a 3D convolutional neural network using an architecture previously described (Gautherot et al., 2021). The weights of the model were determined by minimizing the cost function, here the mean absolute error between chronological age and predicted brain age. To optimize the weights, we used a stochastic gradient descent optimization algorithm (Sutskever et al., 2013) with a learning rate of 0.001, a momentum of 0.1, and a learning rate decay of 5e-05. We used a batch size of 8 during 150 iterations. During the training phase, we performed a data augmentation strategy on-the-fly consisting of performing translation and rotation of the MR images. This technique generated additional artificial training images to prevent the model from overfitting and was empirically found to yield better performance (Shorten and Khoshgoftaar, 2019). We used a 5-fold cross-validation procedure on our training set for optimizing hyperparameters. The mean absolute error of the model on our validation dataset was 4.4 years.

As recommended in BrainAGE studies, we removed any common variance with chronological age before submitting the residualized version of BrainAGE, using linear regression (Liang et al., 2019):


(1) $$\begin{array}{l} \mathrm{Regressed}\;\mathrm{predicted}\;\mathrm{age}=\mathrm{intercept}+\alpha \times \;\mathrm{chronological}\;\mathrm{age}\\ \;+\;\mathrm{error} \end{array}$$

α is the regression coefficient associated with the chronological age, and in our study α = 0.13.

Weights from the pre-trained model were used for the prediction of brain age for our SLE patients and matched controls. BrainAGE was calculated as the difference between predicted brain age and chronological age at the acquisition time, and converted in *z*-score taking the control group as reference (Gautherot et al., 2021).

To understand which brain regions were mainly used by the BrainAGE model, we computed attention maps using an occlusion sensitivity method adapted from Petsiuk et al. (2018). A sliding mask of 8 × 8 × 8 voxels was used to hide part of the input images and probe the model. The final attention map was generated as a linear combination of the binary masks where the combination weights come from the amplitude of the difference between predicted age with and without masking.

### 2.5. Statistical analysis

Statistical analyses were performed using SPSS version 26 (RRID:SCR_002865). Continuous variables are presented as mean (± standard deviation) and categorical variables are presented as numbers (percentage). The normality of the distribution of quantitative variables was assessed visually. Plasma NfL concentrations were log-transformed to satisfy the normality assumption. First, BrainAGE (*z*-score) and brain volumes were compared between controls and SLE patients using a student-*t* test. WMH volumes were compared using a Mann–Whitney *U* test. Then, in order to estimate the risk factors for increased BrainAGE, SLE patients were divided into 2 groups (high vs. low BrainAGE). To define the cut-off value between High and Low BrainAGE, we used the “reaction time” performance. Increased reaction time is one of the main neuropsychological landmarks of brain aging. We performed a ROC-analysis and calculated the Youden Index. The optimal threshold to identify patients with low “reaction time performance” (standardized score < 80) was BrainAGE z-score = 0.9. We therefore used this threshold to separate high and low BrainAGE groups. Group comparisons for biomarkers of neurodegeneration between high and low BrainAGE patients and healthy controls were done using a ANOVA (or Kruskal-Wallis ANOVA). As plasma concentrations of NfL increase with age, group comparisons were adjusted for age using an ANCOVA procedure. We also performed correlation analyses and calculated Pearson correlation coefficient (or Spearman correlation coefficient for non-normally distributed variables) in patients and healthy controls. Finally, to identify the clinical/biological predictors of high BrainAGE, group comparisons between high and low BrainAGE patients were done using a student t-test for continuous variables and a chi-square test for categorical variables. Parameters associated with BrainAGE with a value of *p* <0.2 in univariate analysis were included in a multivariate logistic regression model with BrainAGE as a binary depend variable (high vs. low). To avoid multicollinearity, correlations between variables were checked and variables with r > 0.6 were excluded (disease duration and ongoing prednisolone treatment). A threshold of *p* < 0.05 was used for all statistical significance.

## 3. Results

### 3.1. BrainAGE is significantly increased in SLE patients

As described in material and methods, 70 right-handed female SLE patients and 24 age-matched controls were included in the present study. Table 1 displays the study population details. SLE disease activity (SLEDAI-2 K) and organ damage (SLICC/ACR-DI) scores were low. Sixteen (22.9%) to 22 (31.4%) had a neuropsychiatric presentation according to “SLICC A” and “SLICC B” models, respectively. A detailed description of ongoing medications and neuropsychiatric manifestations is provided in Supplementary Tables S1, S2.

**Table 1:** Clinical, neuropsychological and biological characteristics of the SLE patients.

**Characteristics**	**SLE (*n* = 70)**
**Clinical**	
Age at MRI (y)	35.9 (±9.0)
Disease duration (y)	11.1 (±8.1)
SLICC/ACR-Damage Index	0.7 (±1.1)
SLEDAI-2 K	2.3 (±3.2)
Neuropsychiatric manifestations SLICC A model, *n* (%) SLICC B model, *n* (%)	16 (22.9%) 22 (31.4%)
Renal involvement, *n* (%)†	26 (37.1%)
Smoking (ever), *n* (%)	25 (35.8%)
Prednisolone (ongoing), *n* (%)	55 (78.6%)
Prednisolone daily dose (mg/day)	4.9 (±4.5)
Non-malarial DMARD (ongoing), *n* (%)	41 (58.6%)
Anti-hypertensive drug (ongoing), *n* (%)	21 (30.0%)
**Neuropsychological evaluation**	
Composite memory (standardized score)	95.9 (±6.1)
Psychomotor speed (standardized score)	96.3 (±10.9)
Reaction time (standardized score)	90.3 (±17.4)
Complex attention (standardized score)	96.6 (±22.6)
Cognitive flexibility (standardized score)	96.2 (±21.5)
**Laboratory analyses**	
Log-transformed Plasma NfL (pg/ml)	0.84 (±0.17)
Serum S100A8/A9 (ng/ml)	1.37 (±0.84)
Low complement (ever), *n* (%)	40 (57.1%)
Antiphospholipid antibodies (ever), *n* (%)	22 (31.4%)
Antibodies anti-double stranded DNA (ever), *n* (%)	41 (58.6%)

^†^According to SLICC SLE classification criteria. ACR, American College of Rheumatology; DMARD, disease-modifying antirheumatic drug; NfL, neurofilament light chain; SLE, systemic lupus erythematosus; SLEDAI-2K, Systemic Lupus Erythematosus Disease Activity Index 2000; SLICC, Systemic Lupus International Collaborating Clinics.

**Figure 1:**
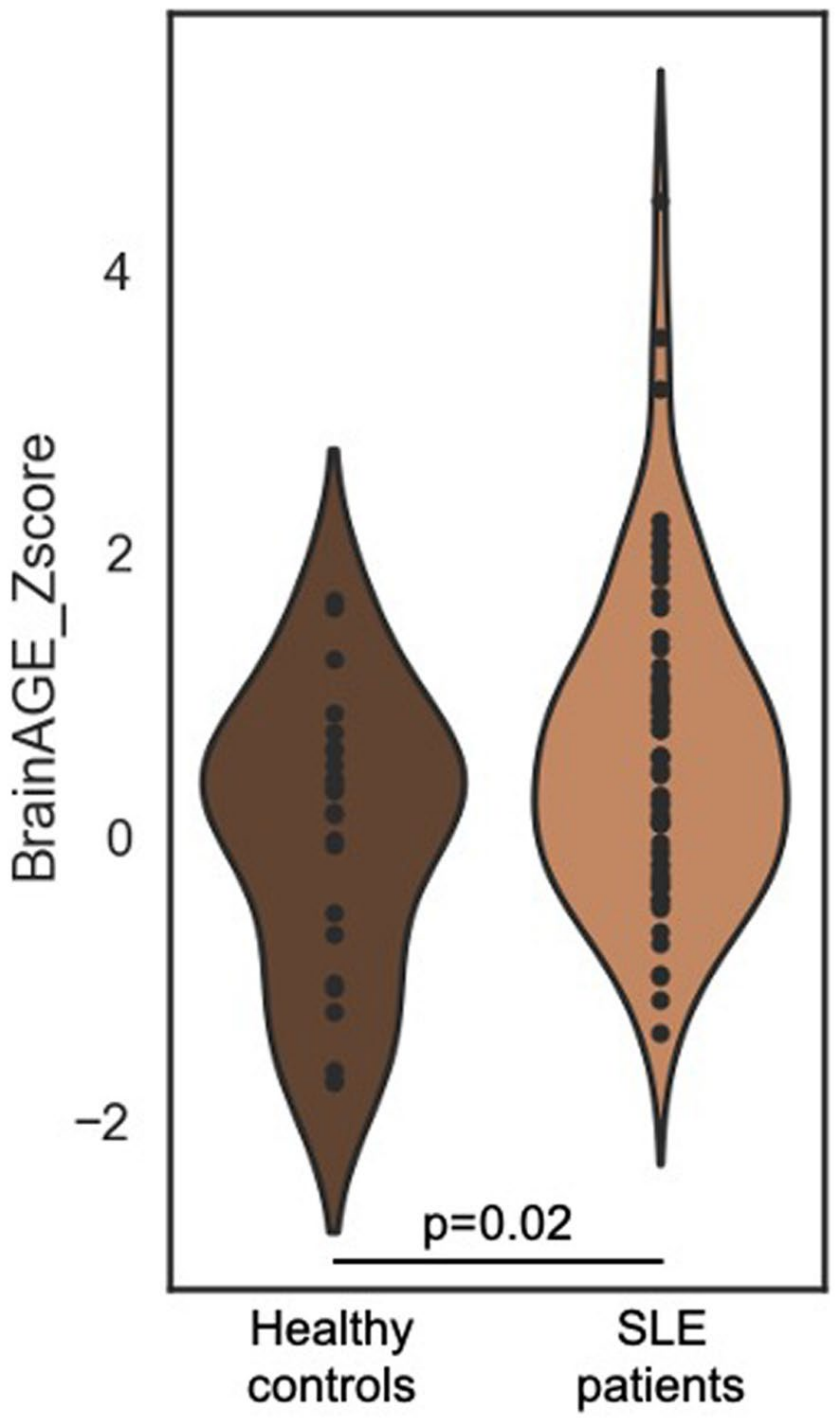
BrainAGE distribution in SLE patients and age-matched healthy controls. Violin plot of BrainAGE (*z*-score) distribution in SLE patients and age-matched controls. Mean BrainAGE is significantly higher in SLE (0.6 [±1.1] vs. 0 [±1], *p* = 0.02). BrainAGE, Brain Age Gap Estimation; SLE, systemic lupus erythematosus.

Mean chronological age of SLE patients and controls was 35.9 (±9.0) and 37.0 (±9.4) years, respectively (*p* = 0.62). BrainAGE z-score was significantly higher in SLE patients, revealing an increased brain aging (0.6 [±1.1] vs. 0 [±1]) (Figure 1 and Table 2). SLE patients had brains looking on average 3.6 years older than age-matched controls. Standard volumetry demonstrated a discrete brain atrophy with slightly increased cerebrospinal fluid volumes in SLE patients (11.96% [±2.50] vs. 10.80% [±2.13], *p* = 0.045) (Table 2). The volume of WMH was modest but significantly higher in SLE patients (0.179 [±0.339] vs. 0.061 [±0.156]). The visual assessment demonstrated no previous ischemic lesion.

**Table 2:** MRI characteristics of SLE patients and comparison with healthy controls.

**MRI characteristics**	**SLE (*n* = 70)**	**Healthy controls (*n* = 24)**	**Cohen’s *d***	**Value of *p***
BrainAGE (*z*-score)	0.6 (±1.1)	0 (±1)	0.56	**0.02**
Gray matter volume (% of total intracranial volume)	51.19 (±2.43)	52.31 (±2.64)	−0.45	0.06
White matter volume (% of total intracranial volume)	36.85 (±1.99)	36.89 (±1.45)	−0.02	0.94
CSF volume (% of total intracranial volume)	11.96 (±2.50)	10.80 (±2.13)	0.48	**0.045**
WMH volume (mL)	0.179 (±0.339)	0.061 (±0.156)	0.39	**0.01**

BrainAGE, Brain Age Gap Estimation; SLE, systemic lupus erythematosus; WMH, white matter hyperintensities. Values of *p* inferior to 0.05 are presented in bold.

**Figure 2:**
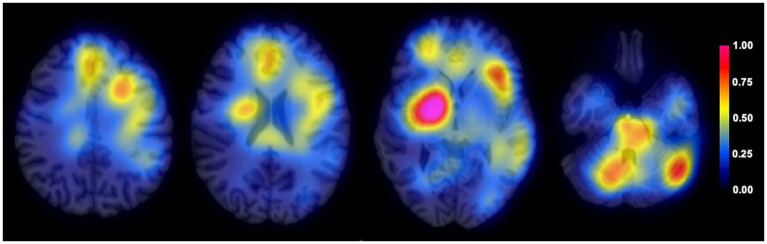
Average attention map for BrainAGE prediction. Average attention map (color-coded) for the entire population (*n* = 94) overlayed on an anatomical T1 image.

Attention maps demonstrated that BrainAGE prediction was mainly based on the following brain areas: right lenticular nucleus, left and right medial prefrontal cortex, left dorso-lateral prefrontal cortex and inferior frontal cortex, posterior part of the body of the corpus callosum, pons, left and right cerebellum hemispheres (Figure 2).

### 3.2. In SLE patients, high BrainAGE is associated with biomarkers of neurodegeneration and poorer cognitive performance

To validate that BrainAGE was reflecting neurodegeneration in SLE patients, we analyzed the association between high BrainAGE (BrainAGE *z*-score > 0.9) and well-established biomarkers of neuronal damage (Table 3). Compared to low BrainAGE and healthy controls, high BrainAGE patients had brain atrophy with increased CSF volume (*p* = 0.001). White matter volume was also lower in high BrainAGE vs. low BrainAGE patients (*p* = 0.02). High BrainAGE patients had higher plasma NfL concentrations after adjusting for age, compared to low BrainAGE patients and healthy controls (*p* < 0.001) (Table 3). WMH volume was higher in high BrainAGE patients compared to healthy controls (*p* = 0.03), but there was no significant difference between high and low BrainAGE patients.

**Table 3:** Comparison of biomarkers of neurodegeneration in high BrainAGE, low BrainAGE SLE patients and healthy controls.

**Variables**	**High BrainAGE (*n* = 24)**	**Low BrainAGE (*n* = 46)**	**Healthy controls (*n* = 24)**	***p-*Value**	**Pairwise** **(adjusted *p* < 0.05)**
BrainAGE (*Z*-score)	1.8 (±0.9)	0 (±0.6)	0 (±1)	**<0.001**	**High BA > Low BA and HC**
**MRI volumes**					
Gray matter volume (% of total intracranial volume)	50.87 (±3.32)	51.35 (±1.84)	52.31 (±2.64)	0.13	NA
White matter volume (% of total intracranial volume)	35.99 (±2.26)	37.31 (±1.70)	36.89 (±1.45)	**0.02**	**Low BA > High BA**
CSF volume (% of total intracranial volume)	13.14 (±2.92)	11.34 (±2.02)	10.80 (±2.13)	**0.001**	**High BA > Low BA and HC**
WMH volume (mL)	0.190 (±0.327)	0.173 (±0.348)	0.061 (±0.156)	**0.03**	**High BA > HC**
**Laboratory markers**					
Age-adjusted log-transformed plasma NfL	0.89 (±0.03)	0.81 (±0.02)	0.70 (±0.03)	**<0.001**	**High BA > Low BA > HC**
**Cognitive performance**					
Composite memory (standardized score)	95.3 (±15.1)	96.2 (±16.8)	100.6 (±12.0)	0.42	NA
Psychomotor speed (standardized score)	91.6 (±9.6)	98.8 (±10.8)	103.5 (±12.4)	**0.001**	**Low BA and HC > High BA**
Reaction time (standardized score)	82.2 (±19.9)	94.6 (±14.3)	93.1 (±21.2)	**0.02**	**HC > High BA**
Complex attention (standardized score)	93.8 (±28.0)	98.1 (±19.3)	105.2 (±8.1)	0.14	NA
Cognitive flexibility (standardized score)	89.0 (±23.9)	100.0 (±19.3)	102.2 (±13.0)	**0.04**	**HC > High BA**

High BrainAGE refers to BrainAGE *z*-score > 0.9. Low BrainAGE refers to BrainAGE *z*-score ≤ 0.9. BA, BrainAGE; BrainAGE, Brain Age Gap Estimation; HC, healthy controls; NfL, neurofilament light chain; SLE, systemic lupus erythematosus. Values of *p* inferior to 0.05 are presented in bold.

Then, we explored the neuropsychological correlates of increased BrainAGE.

**Figure 3:**
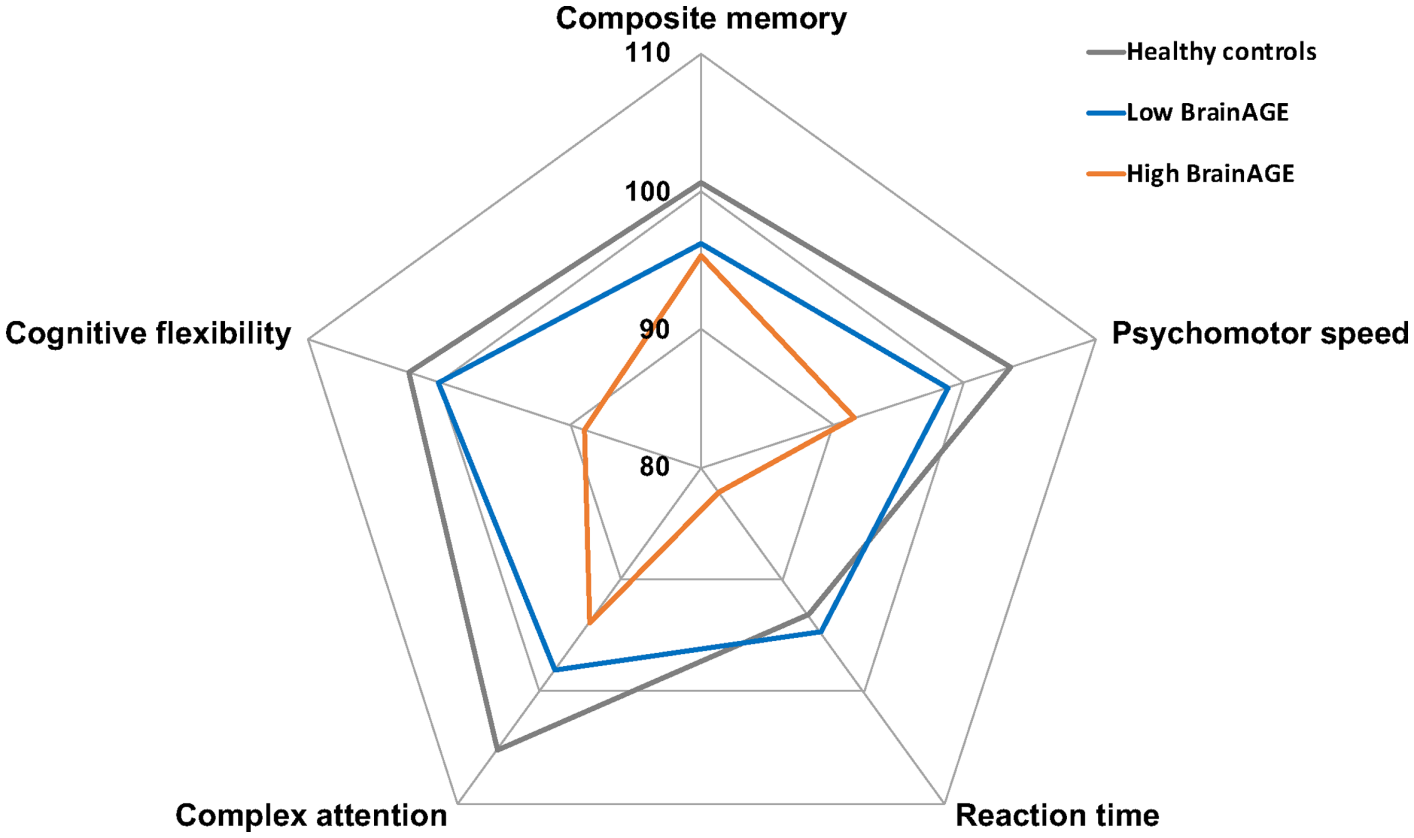
Neuropsychological profile of SLE patients according to BrainAGE. Graphical representation of mean standardized scores in five cognitive domains for healthy controls (gray), high BrainAGE (orange), and low BrainAGE patients (blue). BrainAGE, Brain Age Gap Estimation; SLE, systemic lupus erythematosus.

**Figure 4:**
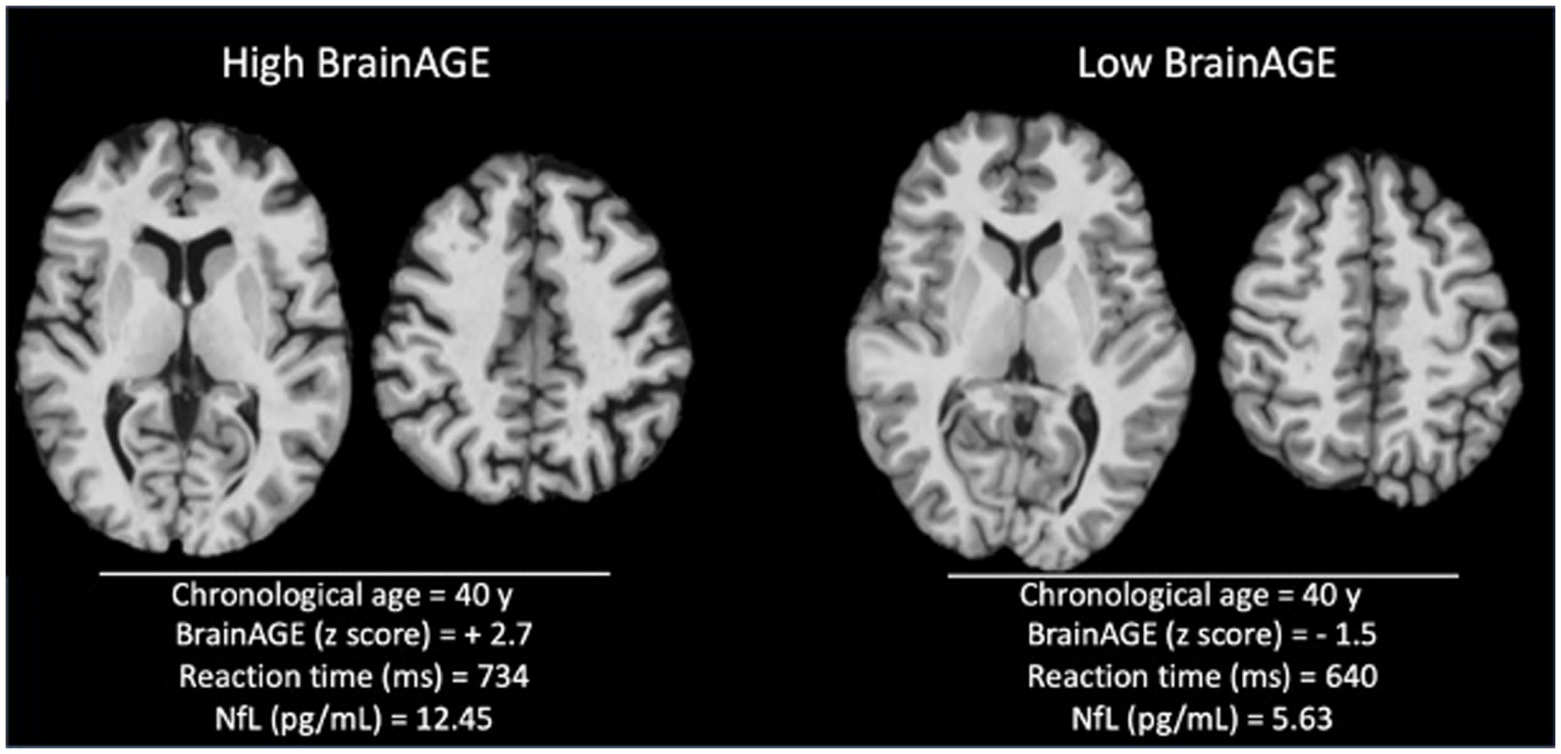
Illustrative cases with high versus low BrainAGE. Skull-striped 3D-T1 images from 40-year patients with high (left) and low BrainAGE (right). Visual inspection demonstrates a higher level of atrophy in the high BrainAGE patient, with enlarged ventricles and sulci. The patient with high BrainAGE has also longer reaction time and higher level of neurofilament light chain (NfL) in plasma.

Neuropsychiatric involvement according to the SLICC attribution models were not more prevalent in high BrainAGE patients (“SLICC A”: 6/24 [25%] vs. 10/46 [21.7%], *p* = 0.76 and “SLICC B”: 8/24 [33.3%] vs. 14/46 [30.4%], 0.80). Nevertheless, SLE patients with high BrainAGE had poorer performance compared to low BrainAGE (BrainAGE *z*-score ≤ 0.9) and/or healthy controls in several cognitive domains (Table 3 and Figure 3): psychomotor speed (*p* = 0.001), reaction time (*p* = 0.02) and cognitive flexibility (*p* = 0.04). Illustrative cases are presented in Figure 4.

Correlation analyses were consistent with the above-mentioned results (Supplementary Table S3). In SLE patients, higher BrainAGE was significantly correlated with lower grey and white matter volume, higher CSF volume, higher level of NfL in plasma, lower cognitive performance for psychomotor speed and reaction time. No significant correlations were found in healthy controls.

### 3.3. Clinical and biological risk factors of high BrainAGE in SLE patients

Finally, we evaluated the association between BrainAGE and the clinical and biological characteristics of disease, to identify the main determinants of increased brain aging (Table 4). In univariate analysis, BrainAGE was associated with some indirect markers of active systemic inflammation. Patients with high BrainAGE were significantly younger at MRI (30.5 [±9.1] vs. 38.8 [±7.5] years, *p* < 0.001), had shorter disease duration (8.4 [±6.8] vs. 12.6 [±8.4] years, *p* = 0.04) and were more likely to have a non-malarial disease-modifying antirheumatic drug (DMARD) (19/24 [79.2%] vs. 22/46 [47.8%], *p* < 0.001). High BrainAGE tended to be associated with higher disease activity (SLEDAI-2K score = 3.1 [±4.2] vs. 1.9 [±2.5], *p* = 0.15), higher prednisolone daily dose (6.1 [±5.6] vs. 4.3 [±3.8], *p* = 0.11) and low complement levels (17/24 [70.8%] vs. 23/46 [50%], *p* = 0.11). We did not find any association between BrainAGE and the presence of anti-phospholipids antibody. In multivariate analysis (Table 5), age at MRI and non-malarial DMARD remained independently associated with BrainAGE (*p* = 0.001 and *p* = 0.01, respectively).

**Table 4:** Clinical/biological characteristics of SLE patients according to BrainAGE group: univariate analysis.

**Variables**	**High BrainAGE (*n* = 24)**	**Low BrainAGE (*n* = 46)**	***P-*value**
**Clinical**			
Age at MRI (y)	30.5 (±9.1)	38.8 (±7.5)	**<0.001**
Disease duration (y)	8.4 (±6.8)	12.6 (±8.4)	**0.04**
SLICC/ACR-Damage Index	0.6 (±1.0)	0.7 (±1.1)	0.77
SLEDAI-2 K	3.1 (±4.2)	1.9 (±2.5)	0.13
Renal involvement, *n* (%)†	11 (45.8%)	15 (32.6%)	0.31
Smoking (ever), *n* (%)	9 (37.5%)	16 (34.8%)	0.64
Prednisolone (ongoing), *n* (%)	21 (87.5%)	34 (73.9%)	0.19
Prednisolone daily dose (ongoing) (mg/day)	6.1 (±5.6)	4.3 (±3.8)	0.11
Non-malarial DMARD (ongoing), *n* (%)	19 (79.2%)	22 (47.8%)	**0.01**
Anti-hypertensive drug (ongoing), *n* (%)	8 (33.3%)	12 (26.1%)	0.56
**Laboratory tests**			
Serum S100A8/A9 (ng/mL)	1.35 (±0.86)	1.38 (±0.84)	0.88
Low complement (ever), *n* (%)	17 (70.8%)	23 (50%)	0.11
Antibodies anti-double stranded DNA (ever), *n* (%)	15 (62.5%)	26 (56.5%)	0.63
Antiphospholipids antibodies (ever), *n* (%)	6 (25%)	16 (34.8%)	0.37

^†^According to SLICC SLE classification criteria. Values of *p* < 0.05 are presented in bold. High BrainAGE refers to BrainAGE *z*-score > 0.9. Low BrainAGE refers to BrainAGE *z*-score ≤ 0.9. ACR, American College of Rheumatology; BrainAGE, Brain Age Gap Estimation; DMARD, disease-modifying antirheumatic drug; SLE, systemic lupus erythematosus; SLEDAI-2K, Systemic Lupus Erythematosus Disease Activity Index 2000; SLICC, Systemic Lupus International Collaborating Clinics.

**Table 5:** Association between BrainAGE and clinical/biological characteristics of SLE patients: multivariate analysis.

**Variables**	***B* coefficient**	**OR**	**95%CI**	***P-*value**
Age at MRI	−0.14	0.87	0.80–0.94	**0.001**
SLEDAI-2 K	0	0.99	0.79–1.26	0.99
Prednisolone daily dose (ongoing)	0.07	1.07	0.91–1.27	0.41
Non-malarial DMARD (ongoing)	1.80	6.03	1.44–25.3	**0.01**
Low complement (ever)	0.43	1.54	0.37–6.34	0.55

Logistic regression model including all variables associated with BrainAGE in univariate analysis with a *p*-value < 0.2. Disease duration and prednisolone ongoing were not included due to multicollinearity. Values of *p* < 0.05 are presented in bold. BrainAGE, Brain Age Gap Estimation; DMARD, disease-modifying antirheumatic drug; SLE, systemic lupus erythematosus; SLEDAI-2K, Systemic Lupus Erythematosus Disease Activity Index 2000.

## 4. Discussion

In this retrospective cross-sectional analysis, we provided evidence of increased brain aging in SLE by applying a deep-learning model to brain MR images. We demonstrated that high BrainAGE was associated with established markers of neurodegeneration and with worse performance in several cognitive domains. Interestingly, higher BrainAGE scores were observed in younger patients and patients with ongoing non-malarial DMARD, suggesting a more active SLE phenotype.

One of the main strengths of our study lies in the use of a deep-learning model to compute a fully automated and time-efficient measurement of brain aging. This approach enables an individualized and non-invasive assessment, which could be implemented into clinical practice. Unlike conventional machine learning models based on brain parcellation and quantification of regional grey matter and white matter volumes, the use of a 3D CNN architecture allowed us to work directly on MR images registered in the MNI space, without introducing any prior assumptions. Processing steps were deliberately limited minimized, using a rigid co-registration method to preserve anatomical details. Notably, our model’s performance to predict the age of healthy controls on the validation sample was in the range of other established models (Franke and Gaser, 2019). This approach has previously demonstrated efficacy in tracking the progression of neurodegeneration in Alzheimer’s disease (Gautherot et al., 2021). Using attention maps, we demonstrated that BrainAGE prediction was based on widespread brain regions where age-related changes have been extensively documented, including the frontal lobe, lenticular nucleus, corpus callosum and cerebellum (Lemaitre et al., 2012; Tullo et al., 2019).

In this study, the utilization of a deep-learning model allowed us to capture brain changes indicative of neurodegeneration in SLE, even within the context of an average low disease activity and subtle differences in brain volumetry between patients and controls. We found that increased brain aging in SLE patients predominantly correlated with a reduction in white matter volume. This observation is consistent with standard MRI analyses showing white matter lesions in 60% of SLE patients with neuropsychiatric symptoms (Jennings et al., 2004). Beyond these clearly apparent lesions, advanced diffusion MRI techniques (such as diffusion tensor imaging) have unveiled widespread alterations even in normal-appearing white matter regions (Kornaropoulos et al., 2022). NfL is another well-established biomarker of axonal damage (Khalil et al., 2018). Lauvsnes et al. (2022) reported a correlation between a loss of cerebral white matter (in the corpus callosum) and plasma NfL concentrations in SLE patients. Similarly, we found a correlation between brain aging and plasma NfL levels.

In our population, increased brain aging in SLE patients resulted in poorer cognitive performance. While SLE patients displaying low BrainAGE had cognitive test scores within the normal range, those with high BrainAGE performed significantly worse in several cognitive domains, reaction time and psychomotor speed being mostly affected. Processing speed is one of the main cognitive domains affected by normal ageing, which has been attributed to a decreased efficiency of interregional communication within the brain (Salthouse, 2016). Indeed, age-related cognitive slowing has been consistently associated with white matter volume reduction, white matter hyperintensities or altered white matter integrity assessed through DTI (Bendlin et al., 2010; Kochunov et al., 2010). Although complex attention was impaired in our SLE patients, in line with a recent meta-analysis (Leslie and Crowe, 2018), the association between complex attention and increased brain aging did not reach statistical significance in our study. Interestingly, neuropsychiatric involvement was not more prevalent in high BrainAGE patients. As already suggested, neuropsychiatric manifestations in SLE may hinge on functional alterations rather than structural modifications (Faust et al., 2010; Steup-Beekman et al., 2013).

Finally, we aimed at identifying the main risk factors of increased brain aging. We found that the clinical phenotype of SLE patients with high BrainAGE was a young subject, with short disease duration, high disease activity and low complement level, treated by non-malarial DMARD and high dose of prednisolone. Drawing from these exploratory results, we can speculate that increased brain age was mainly driven by persistent inflammatory activity under medication. Typically, disease activity in SLE tends to peak early in the disease course and subsequently decline over time (Zhang et al., 2010). Peschken et al. (2019) demonstrated that very high disease activity was associated with shorter disease duration, higher prednisolone dosage and use of DMARD. This initial active phase could be responsible for brain modifications, leading to a disproportionally high BrainAGE in younger patients. A link between chronic peripheral inflammation and modifications of brain structure has been established through murine models of SLE (Chesnokova et al., 2016; Schwartz et al., 2019). The suggested pathway is that ongoing systemic inflammation affects the communication between the peripheral immune system and the brain by activating resident microglial cells. Subsequent abnormal development of neuroprogenitor cells in the corpus callosum and increase neuronal death in the cortex and hippocampus have been observed (Leung et al., 2016; Shi et al., 2018). Direct immunologically mediated neuronal affliction is also involved in neuropsychiatric manifestations of SLE. Anti-NMDA receptor and anti-ribosomal P protein antibodies are considered to target especially the limbic system and are associated with diffuse neuropsychiatric presentations of SLE, while antiphospholipid antibodies are responsible for autoantibody mediated thrombosis and neurovascular manifestations (Schwartz et al., 2019). Of note, the brain changes could be partially reversible under treatment, as illustrated by Mak et al. (2016). Their longitudinal MRI study documented increased brain volume in SLE patients receiving immunosuppressive treatment to mitigate disease activity. We can therefore speculate that BrainAGE scores would decrease over time under a treatment that effectively controls disease activity.

Conversely, we did not find any association between increased brain age and antiphospholipid antibodies or WMH volume. A higher risk of ischemic and hemorrhagic stroke is attributed to SLE with a twofold increase compared to the general population (Wiseman et al., 2016b). Interestingly, ischemic brain changes in SLE patients have been associated with antiphospholipid antibodies (Kaichi et al., 2014; Magro-Checa et al., 2019), independently of SLE activity (Wiseman et al., 2016a). Although BrainAGE is sensitive to vascular lesions (Bretzner et al., 2023), these did not emerge as the main determinants of increased brain aging in our SLE population. The relatively low WMH burden in our population may account for this apparent discrepancy.

The main limitation of our study was its relatively small sample size, which currently curtails the applicability of BrainAGE on an individualized basis and precludes a comprehensive analysis of the intricate interplay between distinct neuropsychiatric manifestations and brain aging. We also acknowledge that the association with disease activity scores was close but did not reach statistical significance. This could be explained by the overall low disease activity observed in our cohort and it warrants validation across populations with a more severe phenotype. Future longitudinal studies will also provide additional insight into the association between age, disease duration and brain aging, as well as the role of glucocorticoids and immunosuppressive treatment.

In conclusion, using a deep-learning BrainAGE model, we provide evidence of increased brain aging in SLE patients, which reflected neuronal damage and cognitive impairment. BrainAGE could be evaluated as an adjunctive diagnostic tool for assessing brain involvement in SLE.

## Data availability statement

The datasets presented in this article are not readily available because the data presented in this study are available from the corresponding author upon reasonable request and approval of the Institutional Review Board. Requests to access the datasets should be directed to pia.sundgren@med.lu.se.

## Ethics statement

The studies involving humans were approved by regional ethics committee in Lund (reference #2012/254, #2012/677, #2014/778). The studies were conducted in accordance with the local legislation and institutional requirements. The participants provided their written informed consent to participate in this study.

## Author contributions

GK: Conceptualization, Formal analysis, Funding acquisition, Methodology, Visualization, Writing – original draft. TR: Data curation, Investigation, Writing – review & editing. KZ: Data curation, Investigation, Writing – review & editing. RL: Formal analysis, Methodology, Software, Writing – review & editing. MG: Methodology, Software, Writing – review & editing. J-PP: Resources, Supervision, Writing – review & editing. AB: Resources, Supervision, Writing – review & editing. OH: Funding acquisition, Resources, Writing – review & editing. AJ: Funding acquisition, Resources, Supervision, Writing – review & editing. PS: Conceptualization, Funding acquisition, Methodology, Project administration, Supervision, Writing – review & editing.

## Footnotes

^1^https://www.applied-statistics.de/lst.html

^3^https://fil.ion.ucl.ac.uk/spm/software/spm12/

^4^https://brain-development.org/

^5^https://www.humanconnectome.org/

^6^https://www.mrn.org/common/cobre-phase-3

^7^https://www.nitrc.org/projects/mcic/

^8^http://schizconnect.org/

^9^http://fcon_1000.projects.nitrc.org/indi/enhanced/

^10^www.ppmi-info.org/data

^11^adni.loni.usc.edu

## Supplementary material

The Supplementary material for this article can be found online at:

https://www.frontiersin.org/articles/10.3389/fnagi.2023.1274061/full#supplementary-material
