# Supplementary material for: MRI BrainAGE demonstrates increased brain aging in systemic lupus erythematosus patients
Source: Front Aging Neurosci. 2023 Oct 20;15:1274061. doi: 10.3389/fnagi.2023.1274061 (PMC10622955; doi:10.3389/fnagi.2023.1274061)
Supplement: Supplementary file 1 [file Table_1.DOCX]

Supplementary Material

# Supplementary Tables

**Supplementary Table 1: Individual neuropsychiatric symptoms in 70 SLE patients**

| **Neuropsychiatric manifestations** | **SLICC A model** | **SLICC B model** |
| --- | --- | --- |
| Any neuropsychiatric manifestation, n (%) | 16 (22.9%) | 22 (31.4%) |
| Cognitive dysfunction, n (%) | 0 (0%) | 5 (7.1%) |
| Depression, n (%) | 0 (0%) | 0 (0%) |
| Autonomic neuropathy, n (%) | 7 (10%) | 8 (11.4%) |
| Cranial neuropathy, n (%) | 7 (10%) | 7 (10%) |
| Cerebrovascular disease, n (%) | 1 (1.4%) | 4 (5.7%) |
| Demyelinating disease, n (%) | 3 (4.3%) | 3 (4.3%) |
| Myelopathy, n (%) | 2 (2.9%) | 3 (4.3%) |
| Acute confusional state, n (%) | 1 (1.4%) | 2 (2.9%) |
| Seizures, n (%) | 1 (1.4%) | 1 (1.4%) |
| Mononeuritis, n (%) | 1 (1.4%) | 2 (2.9%) |
| Aseptic meningitis, n (%) | 1 (1.4%) | 1 (1.4%) |
| Polyneuropathy, n (%) | 1 (1.4%) | 1 (1.4%) |
| Psychosis, n (%) | 1 (1.4%) | 1 (1.4%) |
| Chorea, n (%) | 0 (0%) | 1 (1.4%) |
| Guillain-Barré syndrome, n (%) | 0 (0%) | 0 (0%) |
| Plexopathy, n (%) | 0 (0%) | 0 (0%) |
| Myasthenia gravis, n (%) | 0 (0%) | 0 (0%) |

One patient may have more than one neuropsychiatric manifestation.

Abbreviations: SLE = systemic lupus erythematosus, SLICC = Systemic Lupus International Collaborating Clinics.

**Supplementary Table 2: Ongoing antirheumatic medication**

| **Antirheumatic medication** | **SLE (n=70)** |
| --- | --- |
| Prednisolone, n (%) | 55 (78.6%) |
| Antimalarial medication, n (%) | 55 (78.6%) |
| Non-malarial DMARD, n (%) | 41 (58.6%) |
| - Azathioprin | 22 (31.4%) |
| - Mycophenolate mofetil | 15 (21.4%) |
| - Belimumab | 8 (11.4%) |
| - Intravenous immunoglobulins | 2 (2.9%) |
| - Cyclophosphamide | 1 (1.4%) |
| - Methotrexate | 1 (1.4%) |
| - Rituximab | 1 (1.4%) |

Abbreviations: DMARD = disease-modifying antirheumatic drug, SLE = systemic lupus erythematosus.

**Supplemental Table 3: Correlation of BrainAGE with biomarkers of neurodegeneration in SLE patients and healthy controls**

| **Variables** | **Patients (n=70)**  Correlation coefficient (p value) | **Healthy controls (n=24)**  Correlation coefficient (p value) |
| --- | --- | --- |
| **MRI volumes** |  |  |
| Gray matter volume  (% of total intracranial volume) | -0.31 (**0.004**) | 0.06 (0.39) |
| White matter volume  (% of total intracranial volume) | -0.31 (**0.005**) | -0.16 (0.22) |
| CSF volume  (% of total intracranial volume) | 0.55 (**<0.001**) | 0.16 (0.22) |
| WMH volume (mL) | 0.08 (+/-0.51) | 0.061 (+/-0.156) |
| **Laboratory markers** |  |  |
| Age-adjusted log-transformed plasma NfL | 0.25 (**0.02**) | 0.70 (+/-0.03) |
| **Cognitive performance** |  |  |
| Composite memory (standardized score) | -0.04 (0.37) | -0.32 (0.06) |
| Psychomotor speed  (standardized score) | -0.22 (**0.04**) | -0.06 (0.40) |
| Reaction time  (standardized score) | -0.23 (**0.03**) | 0.17 (0.21) |
| Complex attention  (standardized score) | 0.03 (0.42) | 0.21 (0.17) |
| Cognitive flexibility  (standardized score) | -0.05 (0.34) | 0.16 (0.23) |

P-value calculated for a one-tailed test hypothesis. P-value inferior to 0.05 are in bold.

Abbreviations: BA = BrainAGE, BrainAGE = Brain Age Gap Estimation, HC = healthy controls, NfL = neurofilament light chain, SLE = systemic lupus erythematosus.
